# Generating Minimal Models of H1N1 NS1 Gene Sequences Using Alignment-Based and Alignment-Free Algorithms

**DOI:** 10.3390/genes14010186

**Published:** 2023-01-10

**Authors:** Meng Fang, Jiawei Xu, Nan Sun, Stephen S.-T. Yau

**Affiliations:** 1 Institute for Interdisciplinary Information Sciences, Tsinghua University, Beijing 100084, China; 2 Qiuzhen College, Tsinghua University, Beijing 100084, China; 3 Department of Mathematical Sciences, Tsinghua University, Beijing 100084, China; 4 Yanqi Lake Beijing Institute of Mathematical Sciences and Applications, Beijing 101408, China

**Keywords:** minimal model, longest common sequence, multiple sequence alignment, natural vector, virus tracing

## Abstract

For virus classification and tracing, one idea is to generate minimal models from the gene sequences of each virus group for comparative analysis within and between classes, as well as classification and tracing of new sequences. The starting point of defining a minimal model for a group of gene sequences is to find their longest common sequence (LCS), but this is a non-deterministic polynomial-time hard (NP-hard) problem. Therefore, we applied some heuristic approaches of finding LCS, as well as some of the newer methods of treating gene sequences, including multiple sequence alignment (MSA) and k-mer natural vector (NV) encoding. To evaluate our algorithms, a five-fold cross validation classification scheme on a dataset of H1N1 virus non-structural protein 1 (NS1) gene was analyzed. The results indicate that the MSA-based algorithm has the best performance measured by classification accuracy, while the NV-based algorithm exhibits advantages in the time complexity of generating minimal models.

## 1. Introduction

Influenza is a viral respiratory infection that is usually seasonal in nature. Influenza viruses are divided into types A, B, C and D, each belonging to a genera of the family *Orthomyxoviridae* [1,2]. The hosts and transmissibility of different types of influenza viruses vary, with type A being highly infectious to humans and animals [2]. In March 2009, a type A influenza virus emerged in Mexico and the United States, which was later tagged as H1N1. The name H1N1 stands for the viral type 1 haemagglutinin and type 1 neuraminidase. According to the official report of the World Health Organization [3], it had spread to over 200 countries and regions, resulting in 17,843 deaths by 28 March 2010, while it is estimated that the actual number of deaths far exceeds this number. The origin of the H1N1 influenza virus is believed to be a reassortant virus that had been circulating in pigs, and it is likely that swine-to-human transmission began to occur several months prior to the outbreak [4].

The influenza viral genome consists of segmented negative-strand RNA relying on viral-originated RNA polymerase for replication [1]. A typical type A influenza virion contains eight RNA strands, three encoding the viral RNA polymerase, one encoding the haemagglutinin, one encoding the neuraminidase, one encoding the nucleoprotein which bounds the viral genome, one encoding the matrix protein and membrane protein and one encoding the nuclear export protein and the non-structural protein 1 (NS1) [5]. NS1 is the key toxigenic protein of influenza viruses such as H1N1. It acts by inhibiting post-transcriptional processing of cellular pre-mRNA and shutting off cellular protein synthesis without affecting viral protein synthesis [6]. Mutations in the NS1 gene are associated with H1N1 virulence [7] and has the potential to serve as an indicator of H1N1 evolution.

H1N1 is also believed to be the pathogen of the 1918–1919 pandemic that affected the world, killing an estimated 50–100 million people [8]. Although it turns out that the H1N1 strain prevalent in the 2009 pandemic was less lethal than that in the 1918–1919 pandemic, concerns emerged that subsequent mutations in H1N1 could trigger higher lethality due to the widespread transmission and mutation-prone nature of RNA viruses [9]. Especially with the profound lesson of today’s SARS-nCOV-2 pandemic, the importance of timely tracking of mutations in pandemic viruses is more recognized. In order to properly define the different strains of viruses and determine whether newly identified viruses evolve from a baseline strain, it is helpful to develop “minimal models” of viral genomes. We conceptually define a minimal model of a cluster of virus genomes as a representative benchmark of all viral genome sequences in the cluster. One may define the minimal model of a genome cluster as the longest common subsequence (LCS) of all genome sequences in the cluster, while there can be various definitions corresponding to different clustering algorithms.

To date, only one previous study has analyzed the genome from the perspective of the minimal model, which treats the bacterial genome as the evolution result of the minimal model during random mutation and replication [10]. To the best of our knowledge, there is no known work that uses the minimal model of the genome as a baseline for genome comparison and clustering. Current methods for comparison and clustering of genomes are usually divided into alignment-based methods represented by multiple sequence alignment (MSA) [11] and alignment-free methods represented by natural vector (NV) [12]. In this work, we adopted three distinct algorithms to develop the minimal model of H1N1 viruses in different districts based on their NS1 gene sequences, including two alignment-based methods and one alignment-free method. The first method is to find the LCS of NS1 gene sequences of all viruses in the district to be studied. As is commonly known, there exists a dynamic programming algorithm with $O\left(n^{2}\right)$ complexity to compute the LCS of two sequences, but computing the LCS of multiple sequences is a non-deterministic polynomial-time hard (NP-hard) problem [13]. We thereby conducted some pre-processing on the data and adopted some heuristics. The second method is based on a mature MSA algorithm named Clustal Omega [14]. The third method is on the basis of the alignment-free method. We applied NV encoding to the sequences. NV is a Euclidean space vector representation of gene sequences, which maps gene sequences of different lengths to vectors of the same dimension. NV is widely used in phylogenetic analysis of genetic sequences [12], sequence comparison [15], tracing of emerging pathogens [16] and mutation distribution analysis [17], and it also provides geometric perspective for genome sequence description [18]. NV has the benefit of preserving distances to a certain extent when mapping gene sequences into Euclidean space, allowing for high-speed sequence comparison.

We evaluated these algorithms in a five-fold cross validation classification scheme. First, we regarded viruses from every district as a cluster. Only districts with at least five sequences were retained. Then, 80% of the sequences in every cluster were randomly chosen as the training set to compute the minimal model of each class, while the remaining 20% of sequences were used as the test set. The process was repeated five times, each time using different training set and test set. Finally, the classification accuracy on the test set using the average weighted F1-score scoring index was computed. Through our work, the feasibility of genome classification by minimal models was verified. By the five-fold cross validation scheme, we compared the accuracy of different algorithms. We also compared the time complexity of different algorithms in generating the minimal model and predicting the class of genome. Our results have provided a reference on the choice of algorithms for constructing minimal models of genomes and applying minimal models for genome clustering.

## 2. Materials and Methods

### 2.1. Dataset

A total of 18,211 H1N1 NS1 sequences belonging to 845 districts were retrieved from the National Center for Biotechnology Information [NCBI, https://www.ncbi.nlm.nih.gov (accessed on 12 April 2022)] with keywords “H1N1” and “NS1”, and 3169 sequences from 148 districts were kept. The number of sequences in each district was between 5 and 50. We assumed that viruses in the same district shared similar NS1 sequence features, so the minimal model of viral NS1 sequences in each district could serve as a representative and predictive baseline for tracing viruses with new sequences.

### 2.2. Longest Common Sequence-Based Minimal Model and Distance

To find a minimal model of a group of sequences, the starting point was to find their LCS. Since the multiple sequence LCS problem is NP-hard, there is no polynomial time algorithm to find the LCS at present. In addition, the LCS of a group of sequences is sensitive to outliers: if the features of a sequence are very different from those of other sequences, the resulting LCS will be greatly affected by the sequence. To overcome these problems, a heuristic approach described below was adopted.

For sequences *a* and *b*, we measured their dissimilarity by $\min\left(l\left(a\right),l\left(b\right)\right)-l\left(\mathrm{LCS}\left(a,b\right)\right)$, where $l\left(\cdot \right)$ denotes the length of a sequence, and $\mathrm{LCS}\left(\cdot ,\cdot \right)$ denotes the LCS of two sequences, which can be computed in polynomial time. For a group of sequences with size *M*, we randomly selected 5 sequences from the group and computed the corresponding 5×M matrix *D*. $D\left[i,j\right]$ denotes the dissimilarity between the *i*-th randomly selected sequence and the *j*-th sequence in the sequence group. All sequences whose index *j* satisfied $\left|\left\{i:D\left[i,j\right]>100\right\}\right|\geq 4$ were considered as outliers and were not selected, as we considered these sequences as outliers in the group because at least 4 out of 5 randomly selected sequences have dissimilarities greater than 100 of them. For the remaining sequences, we randomly selected a sequence as the starting point and compared it with the remaining sequences one by one in a random order. Only the LCS of the two comparison sequences were kept at a time. We repeated the one-by-one comparing process five times and took the longest common sequence as the minimal model of the group. This process aimed to eliminate the difference between the LCS generated by one-by-one comparison and the global LCS generated by the random comparison order.

To calculate the distance between two minimal models, the Levenshtein distance (L), which is defined as the minimum number of conversions required to convert one sequence to another, was used as the distance function. Permitted conversions include inserting a character, deleting a character and substituting one character by another. The Levenshtein distance of sequence *a* and sequence *b*, denoted as $L\left(a,b\right)$, can be computed by dynamic programming according to the following recursive formula:

$$\begin{array}{cc} L\left(a\left[0:0\right],b\left[0:0\right]\right)= & 0\\ L\left(a\left[0:m\right],b\left[0:n\right]\right)= & \min\left\{\begin{array}{c} L\left(a\left[0:m-1\right],b\left[0:n\right]\right)+1\\ L\left(a\left[0:m\right],b\left[0:n-1\right]\right)+1\\ L\left(a\left[0:m-1\right],b\left[0:n-1\right]\right)+1_{a\left[m\right]\neq b\left[n\right]} \end{array}\right.,\\ \; & 1\leq m\leq l\left(a\right),1\leq n\leq l\left(b\right). \end{array}$$

Here, $1_{a\left[m\right]\neq b\left[n\right]}$ equals 1 if $a\left[m\right]\neq b\left[n\right]$, otherwise 0; $a\left[i:j\right]$ denotes the sub-sequence between index *i* and index *j* of *a* (including *i* but not *j*; the index is 0-based). For example, if a=“ACGTGCAT”, then $a\left[2:4\right]=“\mathrm{GT}”$. The end of the recursion $L\left(a\left[0:l\left(a\right)\right],b\left[0:l\left(b\right)\right]\right)$ is equal to $L\left(a,b\right)$.

### 2.3. Multiple Sequence Alignment-Based Minimal Model and Distance

**Figure 1:**
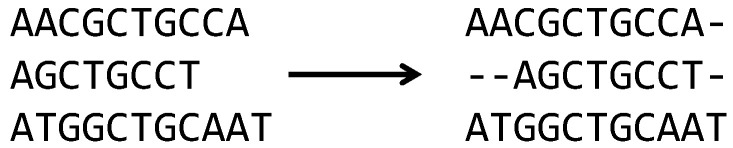
An example of multiple sequence alignment (MSA). There are three original sequences: seq 1: AACGCTGCCA; seq 2: AGCTGCCT; seq 3: ATGGCTGCAAT. By adding “-” in the proper situations, we obtain three aligned sequences: seq 1’: AACGCTGCCA-; seq 2’: -AGCTGCCT-; seq 3’: ATGGCTGCAAT. The lengths of the three sequences are the same, and most positions of the sequences share common bases.

Multiple sequence alignment is usually used in sequence comparison. One of the purposes of MSA is to align multiple biological sequences by filling gaps (“-”) at proper locations in the sequences to generate the minimum number of non-homogeneous sites. An example is given in Figure 1.

There is no precise definition of “minimum number of non-homogeneous sites”, and all MSA algorithms are heuristic. Clustal Omega is one of the latest algorithms which works by first performing a pairwise alignment using a *k*-tuple approach, then a clustering sequence by the mBed method and the *k*-means method, followed by constructing guide trees using the UPGMA method and finally performing a progressive alignment using the HHalign package [19].

In our experiments, we used the Clustal Omega 1.2.4 software package with default parameters to perform MSA, and the minimal model was defined on the basis of the consensus sequence. For each position in the aligned sequence, if a base (A/G/C/T) appeared in at least 50% of the sequences, we appended the base to the corresponding position of the minimal model; otherwise, we dropped the position. For instance, in the example given by Figure 1, the minimal model was “AGCTGCCA”, where the 2nd, 3rd and 11th positions were dropped because no base appears in 50% of the sequences. The same as in the previous subsection, we used the Levenshtein distance *L* to compute the distance of the newly given sequence and the existing minimal model and predict the district of the new gene sequence.

### 2.4. Natural Vector-Based Minimal Model and Distance

The *k*-mer natural vector is an encoding approach to map gene sequences into a Euclidean space [12]; *k*-mer is one of the $4^{k}$ sub-sequences of length *k* with each position chosen from {A, T, G, C}. A gene sequence can be mapped to a $3\times 4^{k}$-dimensional vector. Given a sequence *S*, the first $4^{k}$ entries of the vector are $n_{1},n_{2},\cdots ,n_{4^{k}}$, where $n_{i}$ denotes the number that *k*-mer *i* occurs in the sequence. The second $4^{k}$ entries of the vector are $\mu_{1},\mu_{2},\cdots ,\mu_{4^{k}}$, where $\mu_{i}$ is defined as the arithmetic mean of the distances from all ith *k*-mers to the first base in the sequence. If $n_{i}=0$, $\mu_{i}$ is defined to be zero. The last $4^{k}$ entries of the vector are $\nu_{1},\nu_{2},\cdots ,\nu_{4^{k}}$, where $\nu_{i}$ is defined as the second order normalized central moment of the distances from all *k*-mer *i*’s to the first base in the sequence. Particularly,

$$\nu_{i}=\left\{\begin{array}{cc} 0 & ,\;\mathrm{if}\;n_{i}=0;\\ \sum_{j=1}^{n_{i}}\frac{{\left(s_{ij}-\mu_{i}\right)}^{2}}{n_{i}\left(l-k+1\right)} & ,\;\mathrm{otherwise}. \end{array}\right..$$

Here, *l* is the length of the gene sequence, and $s_{ij}$ is the distance between the *j*th *k*-mer *i* to the first base. Finally, the $3\times 4^{k}$-dimensional vector e, called natural vector (NV), is normalized to have Euclidean norm 1 by dividing each element with the Euclidean norm of the original vector, and we use $e\left(\cdot \right)$ to denote the encoding process:

$$e=e\left(S\right)=\frac{1}{\sqrt{\sum_{i=1}^{4^{k}}\left(n_{i}^{2}+\mu_{i}^{2}+\nu_{i}^{2}\right)}}{\left(n_{1},\cdots ,n_{4^{k}},\mu_{1},\cdots ,\mu_{4^{k}},\nu_{1},\cdots ,\nu_{4^{k}}\right)}^{⊤}$$

One can consider higher order moments to obtain higher dimensional NV encoding, but extending the NV with higher-order moments has proved of little use [12] and thus here we considered up to the second order moment.

For two NVs $e_{1}$ and $e_{2}$, their distance *d* can be calculated by the cosine similarity:

$$d\left(e_{1},e_{2}\right)=1-\frac{1}{∥e_{1}∥∥e_{2}∥}\langle e_{1},e_{2}\rangle$$

where $∥\cdot ∥$ is the Euclidean norm, and $\langle \cdot ,\cdot \rangle$ is the natural inner product. The biological distance of two sequences can be represented by the mathematical cosine similarity of the two corresponding NVs.

The minimal model of a group of sequences based on NV is a “central point” which has a minimal sum of distances to the other sequences’ NVs (in the sense of cosine similarity). This problem has no analytical solution, so we applied an iterative algorithm [20] described as follows. First, the weight of each point is initialized as 1. Then, the weighted mean of all points as the central point is computed and normalizes the central point to Euclidean norm 1 iteratively. The weight of each point is updated to the exponential of their negative distances to the central point. When the distance of the central points computed in adjacent iterations is lower than the given convergence threshold, or the number of iterations reaches a predefined maximum value, the iteration is terminated. The maximum iteration time was set to be 10,000 and the convergence threshold to be $10^{-7}$ in our experiment. This “central point” was considered as the minimal model of the group of sequences. It did not necessarily have a corresponding gene sequence but could still serve as a baseline for comparison with other sequences according to the cosine similarity between NVs.

### 2.5. Five-Fold Cross Validation Classification Scheme

All algorithms (LCS-based, MSA-based and NV-based) were evaluated and compared under the five-fold cross validation classification scheme. We repeated the following steps five times; each step used different test sets. First, we randomly chose 80% of the sequences in each district as the training set to generate a minimal model for that district. Then, we used the remaining 20% of the sequences as the test set. For each sequence in the test set, we first computed the distance between each minimal model and the sequence. The distance computation was based on a properly defined distance function. Then, we predicted that the the sequence belonged to the district whose corresponding minimal model had the minimum distance to the sequence.

Formally, D was used to denote the collection of all 148 districts: $D=\left\{D_{1},D_{2},\cdots ,D_{148}\right\}$. Each district contained $N_{i}$ H1N1 NS1 gene sequences: $D_{i}=\left\{S_{1},S_{2},\cdots ,S_{N_{i}}\right\}$, where $S_{j}$ belongs to the sequence space S, which contains all finite strings consisting of characters “A”, “T”, “G” and “C”. In each fold, a district $D_{i}$ was divided into a training set $D_{i}^{\mathrm{train}}$ and a test set $D_{i}^{\mathrm{test}}$, where $D_{i}=D_{i}^{\mathrm{train}}\cup D_{i}^{\mathrm{test}}$, $D_{i}^{\mathrm{train}}\cap D_{i}^{\mathrm{test}}=\emptyset$ and $\left|D_{i}^{\mathrm{test}}\right|\approx \frac{1}{5}\left|D_{i}\right|$. The collection of subsets of S was denoted as D. Each algorithm applies an encoding function on sequences, which maps a sequence into an encoding space E:

e:S→E.

For the the MSA-based algorithm, the encoding space E is S itself, and the encoding function *e* is just the identity map. For the *k*-mer NV-based algorithm, the encoding space E is the Euclidean space $R^{4^{k}\times 3}$.

The minimal model generating function is an algorithm whose input is a set of sequences and output is an element in the encoding space:

m:D→E.

The output represents the set of sequences to some extent.

The distance function is also an encoding map based on a gene sequence pair:

$$d:E\times E\to R_{+}.$$

The classification function c:S→D is defined based on the distance function:

$$c\left(S\right)={\mathrm{argmin}}_{i}d\left(e\left(S\right),m\left(D_{i}^{\mathrm{train}}\right)\right).$$

If there are multiple districts whose minimal models have the same minimal distance to sequence *S*, $c\left(S\right)$ takes the minimal index among them. The classification performance is measured by the weighted average F1-score:

$$\bar{F1-\mathrm{score}}=\frac{\sum_{i=1}^{\left|D\right|}\left|D_{i}^{\mathrm{test}}\right|\cdot {F1-\mathrm{score}}_{i}}{\sum_{i=1}^{\left|D\right|}\left|D_{i}^{\mathrm{test}}\right|},$$

where

$${F1-\mathrm{score}}_{i}=\frac{2\times P_{i}\times R_{i}}{P_{i}+R_{i}},$$

$$P_{i}=\frac{{\mathrm{TP}}_{i}}{{\mathrm{TP}}_{i}+{\mathrm{FP}}_{i}},$$

$$R_{i}=\frac{{\mathrm{TP}}_{i}}{{\mathrm{TP}}_{i}+{\mathrm{FN}}_{i}},$$

${\mathrm{TP}}_{i}$, ${\mathrm{FP}}_{i}$ and ${\mathrm{FN}}_{i}$ are the true positive number, false positive number and false negative number of the *i*-th class, respectively. Under different criteria, they can be computed differently. Criterion *t* is denoted as saying a sequence is predicted correctly if the minimal model of the district is the *t*-th closest to all districts’ minimal models with respect to the distance function *d*. ${\mathrm{TP}}_{i}$ is the number of correctly predicted sequences in $D_{i}^{\mathrm{test}}$. ${\mathrm{FP}}_{i}$ is the number of sequences *S* satisfying $c\left(S\right)=i$ in ${⋃}_{j\neq i}D_{j}^{\mathrm{test}}$. ${\mathrm{FN}}_{i}$ is the number of incorrectly predicted sequences in $D_{i}^{\mathrm{test}}$. After randomly selecting different training sets $D_{i}^{\mathrm{train}}$ and test sets $D_{i}^{\mathrm{test}}$ each time and repeating all steps 5 times, we computed the arithmetic mean of the 5 weighted average F1-scores as the evaluation of the classification algorithm.

## 3. Results

### 3.1. The Optimal Choice of *k* in the *k*-mer Natural Vector-Based Algorithm

**Figure 2:**
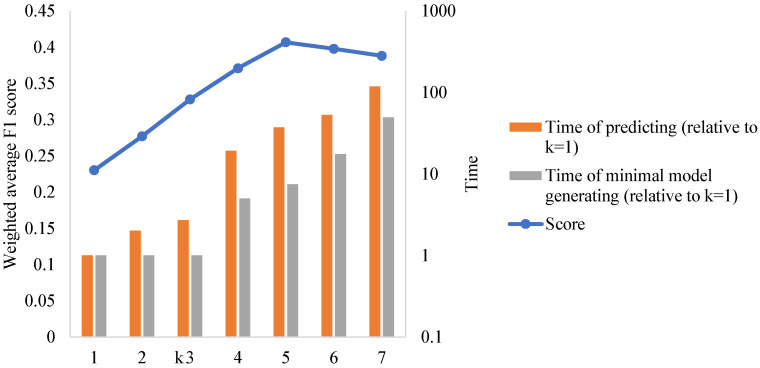
The impact of *k* in *k*-mer natural vector encoding on model performance (criterion 1) and time complexity. The *x*-axis represents the value of k.

The dimension of the *k*-mer NV encoding vectors increases exponentially with *k*, so the parameter *k* determination is of great importance. By performing experiments on other biological problems, the literature study [12] indicated that the optimal choice of *k* lies between $\left\lfloor \log_{4}l_{\min}\right\rfloor$ and $\left\lfloor \log_{4}l_{\max}+1\right\rfloor$, where $l_{\min}$ and $l_{\max}$ denote the minimum and maximum lengths of sequences in the dataset, respectively. In our H1N1 NS1 dataset, the length of most sequences is 660 base pairs, and we observed that $\log_{4}660=4.683$. We also performed the classification tasks using different values of *k*: k=1,2,⋯,7, as displayed in Figure 2, where the x-axis represents the value of *k*. When k=5, the model reaches the optimal accuracy, which agrees with the conclusions of Wen, J. et al. [12]. The two facts show that k=5 is reasonable. In the meantime, when *k* is not greater than three, the model still shows predictive ability in acceptable time complexity.

### 3.2. Multiple Sequence Alignment-Based Algorithm Stands Out in Classification Accuracy

**Figure 3:**
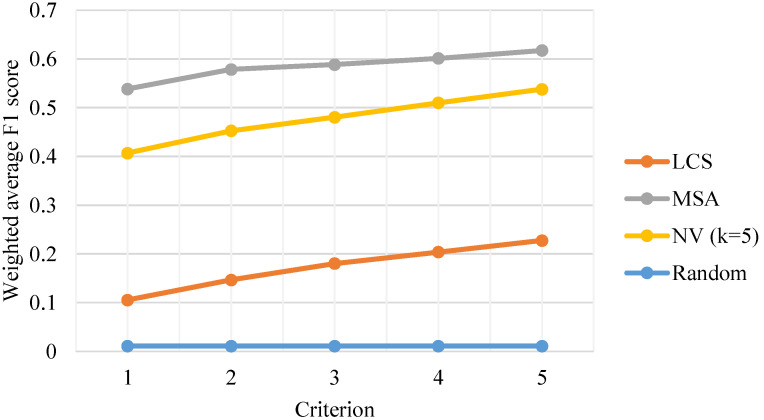
The classification performances comparison of different algorithms measured by weighted average F1-score. LCS: longest common subsequence; MSA: multiple sequence alignment; NV (k=5): 5-mer natural vector.

**Figure 4:**
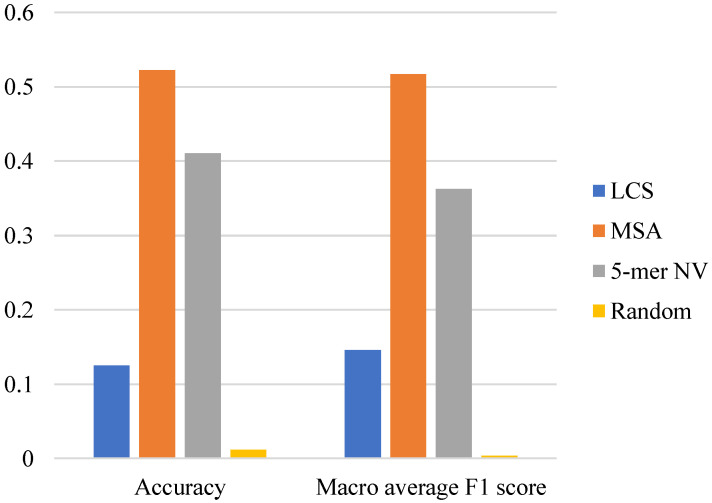
The classification performances comparison of different algorithms based on accuracy and macro average F1-score. LCS: longest common subsequence; MSA: multiple sequence alignment; 5-mer NV: 5-mer natural vector.

The classification performances of the LCS-based, MSA-based and NV-based algorithms were evaluated. The weighted average F1-scores are shown in Figure 3. For the sake of robustness, we chose different criteria to mark the prediction as correct: t=1,2,3,4,5 (see Section 2.5). The “random” model was just to predict the district of each sequence according to the number of sequences in each district’s training set. It can be seen that all algorithms show higher weighted average F1-scores compared with the random model, which means they all have predictive power. Under all criteria, the MSA-based algorithm stood out in classification accuracy. The classification performance comparison among algorithms based on the other performance metrics, including accuracy (the ratio of correct predictions to all predictions) and macro average F1-score (the F1-score averaged on confusion matrices of all classes, see [21] for details), is given in Figure 4.

### 3.3. Natural Vector-Based Algorithm Consumes the Least Time in Generating Minimal Models

**Figure 5:**
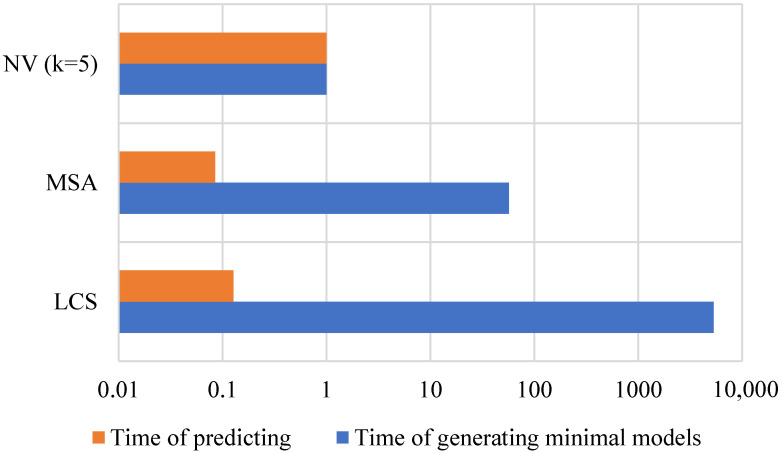
The time complexity comparison of different algorithms. The time complexity is mainly from the minimal model generation and the class of a gene sequence prediction. The x-axis represents the ratio of time consumed by each algorithm to that consumed by the 5-mer NV-based algorithm. LCS: longest common subsequence; MSA: multiple sequence alignment; NV (k=5): 5-mer natural vector.

The time complexity comparison of the three algorithms is shown in Figure 5. The time complexity is mainly from the minimal model generating part and predicting part. The NV-based model consumed the most time of predicting because of the high dimension vectors. The MSA-based and LCS-based models have similar time complexity because they both calculate the Levenshtein distances between a new sequence and all minimal models for prediction. The NV-based algorithm wins in the time of generating minimal models, since the algorithm that generates the “central point” of a group of NVs usually converges in hundreds of iterations.

## 4. Conclusions and Discussion

In this study, we proposed a formal definition and implementation of genome minimal model construction and verified its feasibility for application to genome classification. We applied three algorithms on generating minimal models using the H1N1 NS1 dataset. In order to quantitatively evaluate the minimal models generated by different algorithms, a five-fold cross validation classification scheme was designed. Under this scheme, the LCS-based, MSA-based and NV-based algorithms were tested to generate minimal models and predict the district of newly given sequences. The algorithm based on MSA won in accuracy, while the algorithm based on NV generated the minimal model in the minimum time and maintained predictive power. The *k*-mer NV encoding method involves a parameter *k*, and we reached the same conclusion as Wen, J. et al. did [12] about the best choice of *k* in *k*-mer NV encoding.

Although the NV-based method is slightly less accurate than the MSA-based method in the task of virus district prediction, the former has several advantages that are worth discussing, and some of these advantages are also reflected in other applications of the NV encoding method. Firstly, according to our results of time complexity comparison of generating minimal models and previous studies [22], the NV-based algorithm has higher efficiency compared to alignment-based algorithms. Secondly, the NV-based method can handle genome sequence collections with arbitrary diversity, while MSA-based algorithms can produce potentially unreliable results due to high variability among genomes [23]. Another advantage of the NV-based method is that it can quickly update the minimal model based on newly added sequences. Once a new sequence is detected in a district, it only assigns it an average weight and adds it to the iterative process described in Section 2.4, which can quickly converge to a new NV minimal model representation. As a comparison, the MSA-based method requires re-running the time-consuming MSA algorithm over the entire set of sequences for any sequence joining or change [22]. In addition, since a vector in the continuous Euclidean space does not necessarily correspond to a real sequence in the discrete sequence space, the NV-based method produces a minimal model representation that can achieve finer granularity than the MSA-based method and the LCS-based method.

This work regards the minimal model as a baseline for classification, comparison and virus tracing. Compared to general supervised classification methods, such as the support vector machine (SVM), this method provides better interpretability and renewability because the classification based on the minimal model provides information about the distance between the sequence and the minimal model of each class, and the addition of samples only needs to update minimal models of the involved classes. Moreover, one can generate different minimal models at different classification levels. We only used the H1N1 epidemic district as a classification criterion, while one can also use other classification criteria (for example, the viral subspecies, epidemic period, symptoms caused, etc.) to generate the minimal model of all kinds of virus genomes, and then perform evaluation using the cross validation framework under the hierarchy corresponding to the classification criteria. If there are enough samples for each class, the minimal model can be used to quickly classify viruses with newly discovered genomes and make predictions about any attribute of interest as long as the attribute is a classification criterion for the minimal model generation. Although the NV-based method achieved higher accuracy than the MSA-based method for other tasks, such as phylogenetic analysis [12], the NV-based method is slightly less accurate than the MSA-based method for the task of classifying genomes based on minimal models, implying that the NV-based method is still in the process of being improved. 

## Data Availability

The data presented in this study can be downloaded in the public database, and also available in the Supplementary Materials.

## Supplementary Materials

The following supporting information can be downloaded at: https://www.mdpi.com/article/10.3390/genes1010000/s1, Dataset S1: The H1N1 NS1 gene sequences collected for this work; Code S1: The source code.
